# Incidence of Head Contact Events Including Headers, and Potential Head Injuries at the FIFA Futsal World Cup Lithuania 2021

**DOI:** 10.1111/sms.70083

**Published:** 2025-05-29

**Authors:** Ayda Sarkohi, Alice Hovenberg, Martin Hägglund, Andreas Serner, Kerry Peek

**Affiliations:** ^1^ Medical Faculty, Linköping University Linköping Sweden; ^2^ Unit of Physiotherapy, Department of Health, Medicine and Caring Sciences Linköping University Linköping Sweden; ^3^ FIFA Medical, Fédération Internationale de Football Association Zurich Switzerland; ^4^ Sydney School of Health Sciences, Faculty of Medicine and Health The University of Sydney Sydney New South Wales Australia

**Keywords:** football, futsal, head injuries, heading, soccer, video analysis

## Abstract

Although concern exists regarding the potential relationship between heading, head impacts, and head injuries, and long‐term brain health in association football players, the incidence of these events is yet to be reported in futsal. It has been hypothesized that futsal's format of a smaller field and fewer players might mean that players are exposed to fewer head contact events than those reported in football. Our objective was to analyze the incidence and characteristics of headers, head impacts, and potential head injuries in one men's international futsal tournament. In this cross‐sectional video analysis study, all head contact events (including headers, head impacts, and potential head injuries) from all 52 matches of the FIFA Futsal World Cup Lithuania 2021 were analyzed. There were 1065 head contact events with an incidence rate (IR) of 3014/1000 match hours (MH), including 839 headers (IR2376/1000MH). The most frequent head impact was upper‐limb‐to‐head impacts (*n* = 106, IR300/1000MH) and the least frequent was head‐to‐head impacts (*n* = 8, IR 23/1000MH). There were 38 potential head injuries (IR108/1000MH) primarily from upper‐limb‐to‐head impact. There was a statistically significant relationship between pitch location and type of head contact event (Cramer's *V* 0.18, *p* = < 0.001), with most events occurring outside the penalty areas (83.4%). Headers were the most common head contact event in the FIFA Futsal World Cup, with an IR similar to that reported in football. Although upper‐limb‐to‐head impacts were the primary cause of potential head injuries in both futsal and football, head‐to‐head impacts were less common in futsal.

## 1 Introduction

Futsal is played in over 100 countries with more than 12 million players [1]. Futsal matches are played on a hardcourt with each team being allowed a maximum of five players on the pitch at any one time, with rolling substitution, and a smaller and harder ball, with less bounce than the balls used in association football (hereon in referred to as football). Although injury rates in football and beach soccer have been frequently [2, 3, 4] and recently [5] reported, there are limited such data in futsal. One of the only studies to detail injury incidence at three earlier FIFA Futsal World Cups (2000, 2004, 2008) reported 165 injuries with an incidence rate (IR) of 195.6 injuries per 1000 match hours (MH). The most commonly injured body regions were the lower extremity (*n* = 115, 69.7%, 136.4/1000MH) followed by head and neck (*n* = 21, 12.7%, 24.9/1000MH). The primary mechanism of injury for all injury locations was player‐to‐player contact (*n* = 100, 63.7%, 118.6/1000MH) [6].

Although limited data on injury incidence in futsal exists [6], as well as data related to the performance of futsal skills [7, 8] there are no reported data on heading incidence. Heading is a sport‐specific skill where the head is deliberately used to redirect the ball, which is commonly observed in football [9] and beach soccer [5]. In contrast, futsal is often regarded as a version of football where limited heading occurs [10]. Exposure to head contact events, including heading, head impacts, and/or head injuries, is purportedly associated with a higher risk of neurodegenerative disease in retired football players [11]. However, causal links remain to be established. Knowledge on the incidence of head contact events including heading is an important part in further understanding this relationship. It has been hypothesized that futsal's format with a smaller field and fewer players might expose players to fewer headers than in football [10], similar to heading incidence data reported in other studies using small‐sided games in football [12, 13]. However, no data exist to either confirm or refute this hypothesis.

The main aim of this study was to analyze the incidence and characteristics of head contact events, including headers, head impacts, and potential head injuries in the FIFA Futsal World Cup Lithuania 2021.

## 2 Method

### 2.1 Study Design and Sample

In this cross‐sectional observational video analysis study, head contact events from all broadcasted match footage from the ninth FIFA Futsal World Cup held in Lithuania (12th September—3rd October 2021) were analyzed. There were 24 participating teams, 384 total registered players, and 52 matches. The competition was for men only.

### 2.2 Ethics Statement

An ethics exemption was granted by Swiss Ethics (BASEC‐Nr.: Req‐2023‐01460) since no personal health data were being collected.

### 2.3 Equity, Diversity and Inclusion

Data were collected from the FIFA Futsal World Cup 2021, which is recognized as a men's only competition. Currently no equivalent women's tournament exists; however, the first FIFA Futsal Women's World Cup is due to be held in the Philippines in 2025. The authorship team includes three women (60%) including the lead and last author, as well as a mix of clinical backgrounds (physiotherapy and medicine) and early (including research students) and mid‐ and late‐career stage researchers based across a varied geographical area (Australia, Sweden, Switzerland).

### 2.4 Data Recording Descriptors and Definitions

Head contact events were defined as events where any type of contact was observed to a player's head. Head contact events were further categorized as headers and other non‐heading related events using the definitions in Table 1 [14].

**TABLE 1:** Definitions for head contact events.

Head contact type	Definition
Header	Header: A head‐to‐ball contact where the player makes a deliberate movement to redirect the trajectory of the ball using their head Headers were then further categorized as: – Controlled header: the player shows a level of control when redirecting the ball (such as redirecting the ball toward a goal or teammate), – Uncontrolled header: the player does not display this level of control (for instance when a ball flies into the air or moves in a direction that is in disadvantage to the player and/or team).
Unintentional ball‐to‐head impact	Any physical contact between a player's head and the ball, which is not considered deliberate (e.g., ball impact with a player's face or head when the ball is played at short range)
Other head impacts	Non‐ball related head contacts, including ground‐to‐head, head‐to‐head, upper‐limb‐to‐head, lower limb‐to‐head impact events and other undefined body part‐to‐head impacts.
Potential head injury	An impact to the face or head and the player was unable to resume play for more than 5 s and/or the player was assessed by their medical team. All indirect contact, such as whiplash or rotational movements, to the head/neck were excluded.
Contested nature of the event	All registered head contact events were further categorized as contested or uncontested events: Contested: Two or more opposing players were observed to compete for the ball in close enough proximity that physical contact between players was possible. Contested events were further divided into physical and aerial duels: – Physical duel: opposition players physically competing for a ball while all players have at least one foot on the ground. – Aerial duel: Two or more opposition players competing for a ball that is above shoulder height; where at least one player is off the ground and is being physically challenged by an opposition player Uncontested: only one player is contesting for the ball or has enough space so that there is no possibility for any physical contact with another player

**FIGURE 1:**
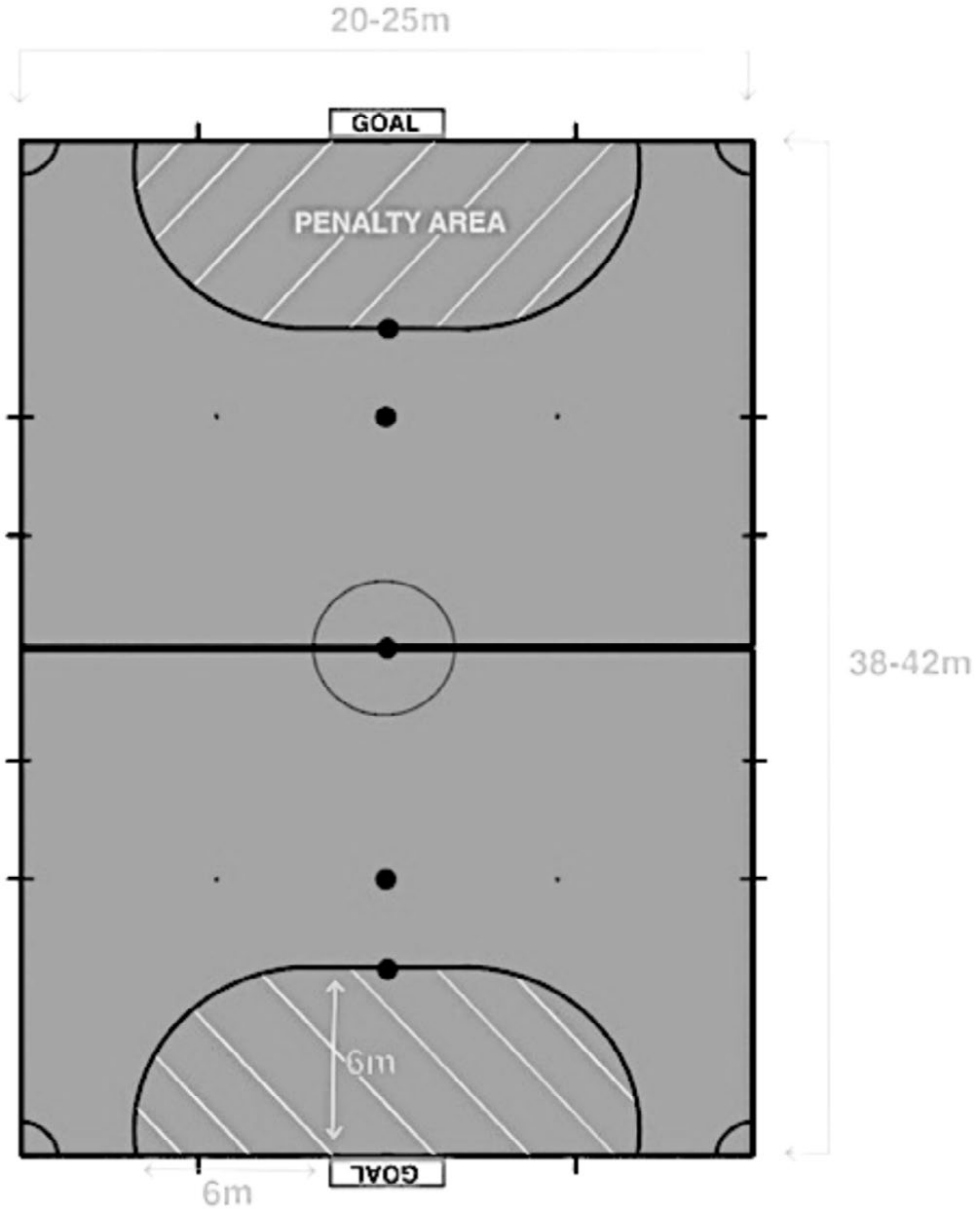
A simplified illustration of a futsal pitch, showing the halfway line which divides the field into two halves and the penalty areas (marked by the white diagonal lines).

Additional data collected for each head contact event included:

- *Match characteristics:* match number, competing teams/countries, final score, score at time of head contact event, period of match when head contact event occurred, match start time on video, time of head contact on video, and time of head contact on match clock.
- *Player characteristics:* team/country of player, player number, position of player (player role), and position on pitch (which is divided into six zones: own penalty area, opposition penalty area, own half, opposition half, middle line and outside pitch, Figure 1) of player with head contact event.
- *Event characteristics:* type of head contact event, whether a header was controlled or uncontrolled, potential head injury, medical assessment of player, immediate substitution of player, presence of physical duel or aerial duel, referee sanction (including foul, yellow or red card).
- *Activity characteristics:* type of activity prior to head contact event such as team possession of the ball prior to the head contact event and ball delivery method, as well as the activity after the head contact event such as retained or gained possession and outcome following head contact event (including potential head injury).

### 2.5 Data Collection

All match videos were provided via the FIFA Football Data Platform. Every match had two video versions, camera 1 (CAM1) and program (PGM) recorded at 24 frames per second. CAM1 was the original footage where most of the pitch was included in the shot, meaning no video replays or footage cuts to coaches and/or single players. PGM was the footage that was broadcast to the public, which included video replays, match timers, and footage cuts to coaches and/or single players. A training session was performed between the project lead (KP‐ a researcher experienced in video coding of head contact events in football) [15] and the first author (ASa), using a prespecified coding sheet in Excel. The first author (ASa) then analyzed all matches by watching the entire CAM1 video and recorded time stamps for all head contact events. Thereafter, the same events were analyzed by ASa in the PGM version and coded according to different characteristics (as per above) into an Excel data collection sheet using those time stamps.

### 2.6 Statistical Analyses

Statistical analyses were performed using Stata 18 (College Station, Texas, USA). Descriptive statistics including counts, means, percentages, and/or IR per 1000 MH are presented for head contact type and other characteristics. IR per 1000 MH was calculated using the following formula ((number of headers ÷ hour of match exposure) × 1000). The match exposure was calculated by using the following equation: number of matches × number of players on the field × duration of the match (in hours). A comparison of types of head contact events and other characteristics (including placement on field among others) between different teams was calculated by using a Chi‐square test (*χ* ^2^) for categorical data with Cramer's *V*, an effect size measurement for the *χ* ^2^. For these, values of 0.15–0.24 and > 0.25 were interpreted as a strong or very strong effect size respectively [16]. Statistical significance for the α‐error was *p* = < 0.05.

Intra‐rater reliability was calculated (by KP) using Cohen's kappa (κ), after the first author (ASa) reanalyzed two randomly selected matches. Cohen's kappa (κ) was interpreted where values ≤ 0 suggest no agreement, 0.01–0.20 (none‐slight), 0.21–0.40 (fair), 0.41–0.60 (moderate), 0.61–0.80 (substantial), and 0.81–1.00 (almost perfect) [17]. A total of 57 head contact events were compared.

## 3 Results

Results of intra‐rater reliability indicated almost perfect agreement except for head contact identification, which was classified as substantial (see Table S1).

### 3.1 Incidence

Across 52 matches there was a total match exposure of 353.3 h containing 1065 head contact events, of which 839 (62.4%, IR 2375/1000MH) were headers (Table 2), with the IR for controlled headers being 1664/1000 MH. A total of 38 potential head injuries were observed, representing 3.6% of all head contact events (Table 2). The IR for potential head injuries were 108/1000MH. Upper‐limb‐to‐head contact (*n* = 23) had the highest IR of 65/1000MH.

**TABLE 2:** Incidence of different head contact events.

Type of head contact event	Count	Incidence rate^a^	Per match^b^		Per player per match^b^	
			Mean	SD	Mean	SD
All head contact events	1065	3014	20.5	±9.1	2.1	±0.9
Headers	839	2375	16.1	±7.8	1.6	±0.8
Controlled headers	588	1664	11.3	±5.7	1.1	±0.6
Uncontrolled headers	186	527	3.6	±2.8	0.4	±0.3
Headers with unsure control	65	184	1.3	±1.2	0.1	±0.1
All head impact events	226	640	4.3	±2.8	0.4	±0.3
Unintentional ball‐to‐head impacts	40	113	0.8	±0.9	0.08	±0.09
Head‐to‐head impacts	8	23	0.2	±0.5	0.02	±0.05
Upper limb‐to‐head impacts	106	300	2.0	±2.0	0.2	±0.2
Lower limb‐to‐head impacts	24	68	0.5	±0.6	0.05	±0.06
Ground‐to‐head impacts	32	91	0.6	±0.8	0.06	±0.08
Undefined head impacts	16	45	0.3	±0.5	0.03	±0.06
Potential head injuries	38	108	0.73	±0.87	0.07	±0.09
Header	2	6	0.04	±0.2	0.004	±0.02
Unintentional ball‐to‐head impacts	4	11	0.08	±0.3	0.008	±0.03
Head‐to‐head impacts	1	3	0.02	±0.1	0.002	±0.01
Upper limb‐to‐head impacts	23	65	0.4	±0.6	0.04	±0.06
Lower limb‐to‐head impacts	4	11	0.08	±0.3	0.008	±0.03
Ground‐to‐head impacts	3	9	0.06	±0.2	0.006	±0.02
Undefined head impacts	1	3	0.02	±0.1	0.002	±0.01

^a^ Incidence rate per 1000 match hours.

^b^ Mean per match and per player per match was calculated based on the number of players on the pitch.

### 3.2 Other Descriptors

#### 3.2.1 Head Contact Events

Frequency and percentage of head contact events per location on pitch, ball delivery method, match outcome after event, duels, and player roles are presented in Tables 3 and 4. There was a statistically significant relationship between pitch location and type of head contact event (Cramer's *V* 0.19, *p* = < 0.001). The most common site for head contact events was outside the penalty areas with 888 events (83.4%), 621 (63.8) being in the player's own half. There was also a statistically significant relationship between ball delivery method and head contact type (Cramer's *V* 0.18, *p* = < 0.001) with free play being the most frequently observed method of delivering the ball (*n* = 783, 58.3%) and the second most common method being goalkeeper's kick (*n* = 233, 17.3%).

**TABLE 3:** Player and event characteristics of different head contact events.

Type of head contact event	Location of pitch—*n* (%)						Player roles—*n* (%)		Duels—*n* (%)			Referee sanction^a^—*n* (%)
	Own half (excluding penalty area)	Opposition half (excluding penalty area)	Own penalty area	Opposition penalty area	Midline	Outside pitch	Outfield player	Goalkeeper	Aerial	Physical	No duel	Yes
All head contact events	621 (58.3)	255 (23.9)	106 (10.0)	71 (6.7)	9 (0.8)	3 (0.3)	1014 (95.2)	51 (4.8)	127 (11.9)	121 (11.4)	817 (76.7)	57 (5.4)
Headers	535 (63.8)	178 (21.2)	73 (8.7)	43 (5.1)	9 (1.1)	1 (0.1)	809 (96.4)	30 (3.6)	102 (12.2)	17 (2.0)	720 (85.8)	4 (0.5)
Controlled headers (*n* = 588)	391 (66.5)	118 (20.1)	46 (7.8)	26 (4.4)	6 (1.0)	1 (0.2)	560 (95.2)	28 (4.8)	62 (10.5)	6 (1.0)	520 (88.4)	1 (0.2)
Uncontrolled headers (*n* = 186)	103 (55.4)	47 (25.3)	21 (11.3)	12 (6.5)	3 (1.6)	—	185 (99.5)	1 (0.5)	34 (18.3)	6 (3.2)	146 (78.5)	3 (1.6)
Indeterminable (*n* = 65)	41 (63.1)	13 (20.0)	6 (9.2)	5 (7.7)	—	—	64 (98.5)	1 (1.5)	6 (9.2)	5 (7.7)	54 (83.1)	—
All head impacts	86 (38.1)	77 (34.1)	33 (14.6)	28 (12.4)	—	2 (0.9)						
Unintentional ball‐to‐head impacts (*n* = 40)	12 (30.0)	9 (22.5)	14 (35.0)	5 (12.5)	—	—	30 (75.0)	10 (25.0)	4 (10.0)	2 (5.0)	34 (85.0)	—
Head‐to‐head impacts (*n* = 8)	4 (50.0)	4 (50.0)	—	—	—	—	8 (100.0)	—	—	8 (100.0)	—	—
Upper limb‐to‐head impacts (*n* = 106)	48 (45.3)	36 (34.0)	5 (4.7)	15 (14.2)	—	2 (1.9)	104 (98.1)	2 (1.9)	19 (17.9)	62 (58.5)	25 (23.6)	31 (29.2)
Lower limb‐to‐head impacts (*n* = 24)	7 (29.2)	8 (33.3)	6 (25.0)	3 (12.5)	—	—	19 (79.2)	5 (20.8)	1 (4.2)	7 (29.2)	16 (66.7)	6 (25.0)
Ground‐to‐head impacts (*n* = 32)	10 (31.3)	16 (50.0)	3 (9.4)	3 (9.4)	—	—	31 (96.9)	1 (3.1)	1 (3.1)	16 (50.0)	15 (46.9)	11 (34.4)
Other head impacts (*n* = 16)	5 (31.3)	4 (25.0)	5 (31.3)	2 (12.5)	—	—	13 (81.3)	3 (18.8)	—	9 (56.3)	7 (43.8)	5 (31.3)

^a^ Referee sanction includes first yellow card, second yellow card, and straight red card given by referee.

**TABLE 4:** Ball delivery method in the different head contact events.

Type of head contact event	Corner	Ball delivery method—*n* (%)								
		Free kick	Free play	Goalkeeper block	Goalkeeper kick	Goalkeeper throw	Kick in	Shot on goal (on target)	Shot on goal (off target)	No ball involved
All head contact events	41 (3.8)	8 (0.8)	655 (61.5)	24 (2.3)	83 (7.8)	157 (14.7)	61 (5.7)	9 (0.8)	4 (0.4)	21 (2.0)
Headers	42 (1.4)	5 (0.6)	486 (57.9)	19 (2.2)	77 (9.2)	146 (17.4)	60 (7.2)	3 (0.4)	1 (0.1)	—
Controlled headers	22 (3.7)	2 (0.3)	356 (60.5)	15 (2.6)	54 (9.2)	96 (16.3)	42 (7.1)	—	1 (0.2)	—
Uncontrolled headers	13 (7.0)	2 (1.1)	92 (49.5)	4 (2.2)	20 (10.8)	40 (21.5)	12 (6.5)	3 (1.6)	—	—
Headers with unsure control	7 (10.8)	1 (1.5)	38 (58.5)	—	3 (4.6)	10 (15.4)	6 (9.2)	—	—	—
All head impacts	1 (0.4)	3 (1.3)	169 (74.8)	5 (2.2)	6 (2.7)	11 (4.9)	1 (0.4)	6 (2.7)	3 (1.3)	21 (9.3)
Unintentional ball‐to‐head impacts	1 (2.5)	1 (2.5)	27 (67.5)	4 (10.0)	1 (2.5)	2 (5.0)	—	4 (10.0)	—	—
Head‐to‐head impacts	—	—	6 (75.0)	—	—	2 (25.0)	—	—	—	—
Upper limb‐to‐head impacts	—	2 (1.9)	79 (74.5)	—	4 (3.8)	5 (4.7)	1 (0.9)	—	1 (0.9)	14 (13.2)
Lower limb‐to‐head impacts	—	—	18 (75.0)	1 (4.2)	1 (4.2)	—	—	2 (8.3)	1 (4.2)	1 (4.2)
Ground‐to‐head impacts	—	—	26 (81.3)	—	—	2 (6.3)	—	—	1 (3.1)	3 (9.4)
Other head impacts	—	—	13 (81.3)	—	—	—	—	—	—	3 (18.8)

Across the entire tournament there were 57 fouls called for head contact events, most commonly for upper‐limb‐to‐head contact (*n* = 31), with only 6 related to headers. Twenty (33.9%) head contact events led to a yellow card, with one straight red card given for a lower limb‐to‐head contact.

There was a statistically significant relationship between the presence of an aerial duel and head contact type (Cramer's *V* = 0.36, *p* = < 0.001) as well as a physical duel and head contact type (Cramer's *V* = 0.65, *p* = < 0.001). A higher proportion of aerial duels and physical duels were observed for head contact events where player‐to‐player contact was observed, such as upper‐limb to head impacts (76.4%), head‐to‐head impacts (100%) as well as and ground‐to‐head impacts (53.1%), than for headers (14.2%) and unintentional ball‐to‐head impacts (15%).

Finally, a higher exposure for outfield players was recorded across all head contact events, representing 95.4% (*for noting* if the risk of head contact was equal across all playing positions, the exposure ratio of outfield players was expected to be 80%), Cramer's *V* 0.22, *p* = < 0.001 (Table 3).

#### 3.2.2 Potential Head Injuries

Similar patterns were observed for match characteristics of potential head injuries (See Table S2). For the location of pitch, most potential head injuries occurred outside the penalty areas compared to other pitch locations; although this relationship was not significant (Cramer's *V* 0.09. *p* = 0.06). For ball delivery method (Cramer's *V* 0.39, *p* = < 0.001), 71.1% of potential head injury events occurred during free play. As for match outcome, there was a relationship between potential head injury events and a referee sanction (Cramer's *V* 0.46, *p* = < 0.001). In total, 16 potential head injury events (42.1%) resulted in a referee sanction, with upper limb‐to‐head impact being the most common contact type (*n* = 11) while ground‐to‐head had the highest proportion of sanction, with all 3 (100%) of ground‐ to‐head impacts resulting in a foul. In addition, all three ground‐to‐head impacts resulted in a yellow card being given to the opposition player, compared with only 21% (*n* = 4) of upper‐limb‐to‐head contact events. The red card given for lower‐limb‐to‐head contact was also considered a potential head injury.

Potential head injuries caused by upper‐limb‐to‐head impact (74.9%), head‐to‐head impact (100%) and ground‐to‐head impacts (100%) were more likely to involve an aerial or physical duel than headers (50%) or unintentional ball‐to‐head impact (25%), although the number of events is small (Table S1).

When observing the player roles and potential head injuries, most were outfield players (*n* = 33, 86.8%), with five events involving goalkeepers (two for lower limb‐to‐head impact, two for unintentional ball‐to‐head impact, and one for other [torso‐to‐head impact], Cramer's *V* 0.06, *p* = 0.04 [Table S3]). In total, 12 (31%) of potential head injury events (IR 36.8/1000MH) were assessed by medical teams (five for upper‐limb‐to‐head impact, three for unintentional ball‐to‐head impact, two for lower limb‐to‐head impact, one for head‐to‐head impact, one for other). The IR of potential head injuries that received on‐or off‐pitch medical assessment was 37/1000 MH. Eleven players were also immediately substituted following the potential head injury event (four for upper‐limb‐to‐head impact, three for unintentional ball‐to‐head impact, four for lower limb‐to‐head impact, two for ground‐to‐head impact, one for head‐to‐head impact).

## 4 Discussion

The main findings of this observational study of all head contact events from the FIFA Futsal World Cup Lithuania 2021 were: first, headers were the most common head contact type, followed by upper‐limb‐to‐head impacts, with relatively few potential head injuries. Second, more headers occurred during free play, particularly outside the penalty areas and more often in uncontested scenarios involving outfield players. Finally, the most common mechanism of potential head injuries was from upper‐limb‐to‐head impact, with this mechanism also resulting in more referee sanctions than any other head contact situation (although proportionally more ground‐to‐head impacts resulted in a referee sanction, although these events were much smaller in number).

When compared to earlier studies in football, the IR of headers (2375/1000 MH) in our study in futsal is similar to the IR of 2509/1000 MH reported in a recent study of headers from the 2018 FIFA World Cup [9] but higher than the IR reported in under‐18 men's (IR 1864/1000 MH) and under‐20 men's football (IR 1761/1000 MH) [15]. These comparisons indicate that the IR of headers in futsal could be on par with football, and therefore does not appear to support a hypothesis that futsal players would be exposed to fewer headers [10]. Although the recorded mean number of headers per player per match in our study (1.6 ± 0.8) are less than the 2018 FIFA World Cup (4 headers) [9], this result in futsal is confounded by shorter matches (40 min compared to 90) [18], therefore the time interval between headers might still be similar. On the other hand, the match clock in futsal stops continuously (which it does not in football), therefore, the total time that players are on the pitch for is underestimated in futsal compared to that of football, which will directly affect the IR calculations and confound any direct comparison with football. When exploring differences in heading exposure between futsal and football it is also important to explore other factors that might influence any direct comparison particularly when considering heading burden (i.e., frequency and head impact magnitude). For instance, futsal is played on a smaller pitch size, therefore the number of headers from the types of long ball deliveries often observed in football (such as goal kicks and corners) would be lower. Headers from corners and free kicks are considered ‘high‐force’ deliveries and have been shown to result in higher head impact magnitudes when compared to headers during free play and throw‐ins [19, 20]. The effect of futsal ball properties on head impact magnitude is also yet to be explored which could be an important consideration given that a futsal ball is smaller yet weighs approximately the same as the ball used in football [21, 22]. The effect of different construction materials between balls is something which should also be considered in the overall assessment of heading burden. Overall, our study findings do not support that the IR of headers in men's futsal are less than those previously reported in men's football.

Twice as many headers and head impact events were observed in the player's own half rather than the opponent's half, despite the number of head contact events being similar between penalty areas. This result might indicate that players playing in defensive roles in futsal may be exposed to higher numbers of head contact events, a finding commonly observed in football [9]. Aerial and physical duels were more commonly observed for head impacts and potential head injuries involving player‐to‐player contact, whereas a much higher proportion of headers (85.8%) were completed without a duel. This finding in headers is different than the number of uncontested headers observed in a sample of matches from the FIFA World Cup 2022, where 67.7% of headers involved no duel [14].

Outfield players in our study received higher numbers of all head contact types when compared with goalkeepers, representing 95.3% of all head contact events and 96.4% of all headers. This finding is consistent with previous research in men's football, where goalkeepers performed < 1% of all headers across the 2018 FIFA World Cup and men's youth under‐18 football within Australia and India [9]. Even if we consider the ratio of outfield players to goalkeepers (4:1) outfield players experienced more headers and other head contact events. However, when exploring potential head injury events caused by unintentional ball‐to‐head and lower limb‐to‐head impacts, 50% of these events were observed in goalkeepers (although these events were low in number which likely confounds this finding). It appears logical that proportionally, goalkeepers would be involved in more lower limb‐to‐head contact events, as they commonly go to ground when attempting to save an incoming shot on goal, thus increasing their likelihood of contact with another player's lower limb. However, a higher proportion of unintentional ball‐to‐head impacts appears less logical, as goalkeepers should be able to protect their head with their hands. It is possible that this result is due to goalkeepers spreading their arms wide when in close proximity to goal situations to cover as much of the goal area as possible. It would be interesting to explore this finding in more detail in future studies in futsal.

There was a total of 38 potential head injuries recorded (IR of 108/1000 MH). A study of three futsal World Cups (2000, 2004, 2008) reported 21 diagnosed injuries to the head or neck area (IR 25/1000 MH) [6]. The difference in injury rates between this earlier study and our study is likely attributed to the difference in coding methodology. The earlier study defined injuries as “any physical complaint during a match which received medical attention from the team physician, regardless of the consequences with respect to absence from matches or training.” [6] Although our study defined potential injuries as: face or head contact where the player is unable to resume play for > 5 s and/or they were medically assessed. If we only compare the number of potential head injuries in our study that received on or off‐pitch medical assessment, this gives an IR of 37/1000 MH, which is similar to the IR reported from the three earlier futsal World Cups. However, it should be noted that none of the events in our study are verified head injuries due to lack of access to injury‐confirming data which means that the IR of verified head injuries in our study could be lower than the number of medically assessed potential head injuries reported. One recently published study from the FIFA World Cup 2022 which used the same definition of a potential head injury, reported an IR of 69/1000 MH potential head injuries [23] which is lower than the 108/1000 MH in our study in futsal. Additionally, when comparing medically assessed potential head injuries there was an IR of 16/1000 MH in football [23] compared to 37/1000 MH in futsal, indicating that while potential head injury data are relatively low in both sports in men, futsal generally has slightly higher rates when using comparable definitions.

In our study, upper‐limb‐to‐head impacts (*n* = 23, 61%) were the most common cause of potential head injuries, with an IR of 65/1000MH which is consistent with earlier studies in men's professional football [4, 24]. Only two headers were categorized as a potential head injury event, which is only 0.19% of total head contact events observed while upper‐limb‐to‐head impacts represented 2.2%. Upper limb‐to‐head impacts also resulted in the highest number of fouls. One study in men's professional football reported a 29% reduction in head injuries from upper‐limb‐to‐head contact following the implementation of a rule to sanction players with a red card where this contact is deemed intentional [2]. These findings highlight the important role that rules changes and referees can play in head injury prevention.

### 4.1 Study Limitations

This study only looked at data from the FIFA Futsal World Cup, a tournament that only provides a sample in professional men at the highest playing level. Therefore, our results may have limited generalizability to other cohorts and skill levels of players such as children/youth, women, and amateur players. In addition, these data cannot be extrapolated to represent the incidence of head contact events across a playing season (which will include both training and matches). Regarding head injuries, no other sources than the match videos were available, meaning the number of actual or verified head injuries is unknown.

## 5 Conclusion

There were similar rates of head contact events in futsal when compared with previously reported data in football. Headers were the most common head contact event type in the FIFA Futsal World Cup Lithuania 2021, with an IR on par with those reported in football. Most players exposed to head contact events as well as potential head injuries were outfield players, with upper‐limb‐to‐head impacts being the primary cause of potential head injuries in futsal. These findings may assist in guiding future research, particularly related to the development of futsal‐specific head injury prevention initiatives.

### 5.1 Perspectives

This study provides valuable insights into the incidence and characteristics of head contact events, including headers and those resulting in a potential head injury in men's futsal. Our findings indicate that most head contact events occur outside the penalty areas, with free play being the most common ball delivery method. Outfield players had a higher exposure to head contact events when compared with goalkeepers. Further, our data suggest that players in defensive roles may be exposed to a higher incidence of head contact events due to the higher number of events in their own half. Similar rates of head contact events were observed in futsal compared to previously reported data in football. Collectively, these findings underscore the need for continued monitoring of head contact events in futsal and may provide guidance on preventive measures to mitigate the risk of head injuries in futsal. For instance, potential head injuries were mostly caused by upper‐limb‐to‐head contact, with a significant proportion resulting in referee sanctions, highlighting the importance of exploring rule changes and supporting referee action in head injury prevention, as evidenced by the reduction in head injuries following the implementation of stricter sanctions for intentional upper‐limb‐to‐head contact in football.

## Author Contributions

**Ayda Sarkohi:** methodology, data curation, formal analysis, visualization, writing – original draft. **Alice Hovenberg:** methodology, data curation, writing – review and editing. **Martin Hägglund:** methodology, writing – review and editing. **Andreas Serner:** conceptualization, methodology, writing – review and editing. **Kerry Peek:** conceptualization, methodology, project administration, data curation, formal analysis, visualization, writing – review and editing. guarantor.

## Disclosure

Patient and Public Involvement: No members of the public were involved in the development of this project due to its observational study design and the inclusion of all players and all types of head contact.

## Ethics Statement

Ethics exemption was granted for this study by the Swiss Association of Research Ethics Committees, Kanton Zurich (BASEC‐Nr.: Req‐2023‐01460).

## Conflicts of Interest

K.P. and A.Se. declare employment with FIFA. All authors declare no other relevant financial or nonfinancial competing interests.

## Supporting information


Data S1

## Data Availability

Data are available upon reasonable request from KP (kerry.peek@fifa.org).
