# Supplementary material for: Incidence of Head Contact Events Including Headers, and Potential Head Injuries at the FIFA Futsal World Cup Lithuania 2021
Source: Scand J Med Sci Sports. 2025 May 29;35(6):e70083. doi: 10.1111/sms.70083 (PMC12122905; doi:10.1111/sms.70083)
Supplement: Supplementary file 1 — Data S1 [file SMS-35-e70083-s001.docx]

Supplementary Table 1: Results for intra-rater reliability

| **Descriptor** | **Cohen’s Kappa (κ)*** |
| --- | --- |
| Head contact identification | 0.70 |
| Head contact type | 0.92 |
| Position of player with head impact | 1.00 |
| Position of pitch when head impact occurred | 0.98 |
| Controlled header | 0.88 |
| Potential head injury event | 1.00 |
| Physical duel | 0.98 |
| Aerial duel | 0.98 |
| Possession status at time of head impact event | 0.94 |
| Possession status following head impact event | 0.98 |
| Ball delivery method | 0.94 |
| Outcome following head impact event | 0.90 |

***** κ values ≤ 0 suggest no agreement, 0.01-0-20 (none-slight), 0.21-0.40 (fair), 0.41-0.60 (moderate), 0.61-0.80 (substantial) and 0.81-1.00 (almost perfect).

Supplementary table 2. Player and event characteristics of different potential head injuries

| **Type of head contact event** | | **Location of pitch- n (%)** | | | | | | **Player roles- n (%)** | | **Duels- n (%)** | | | **Referee sanction* - n (%)** |
| --- | --- | --- | --- | --- | --- | --- | --- | --- | --- | --- | --- | --- | --- |
|  | | **Own half (excluding penalty area)** | **Opposition half (excluding penalty area)** | **Own penalty**  **area** | **Referee sanction* - n (%)** | **Midline** | **Outside pitch** | **Outfield player** | **Goalkeeper** | **Aerial** | **Physical** | **No duel** | **Yes** |
| **All potential head injuries** | | **17**  **(44.7)** | **11**  **(28.9)** | **5**  **(13.2)** | **4**  **(10.5)** | **-** | **1**  **(2.6)** | **33**  **(86.8)** | **5**  **(13.2)** | **6**  **(15.8)** | **16**  **(42.1)** | **16**  **(42.1)** | **16**  **(42.1)** |
| - Total header | | 1  (50.0) | 1  (50.0) | - | - | - | - | 2  (100.0) | - | 1  (50.0) | - | 1  (50.0) | 1  (50.0) |
| - *Controlled headers* | | - | 1  (100.0) | - | - | - | - | 1  (100.0) | - | 1  (100.0) | - | - | 1  (100.0) |
| - *Uncontrolled headers* | | 1  (100.0) | - | - | - | - | - | 1  (100.0) | - | - | - | 1  (100.0) | - |
| - *Headers with unsure control* | | - | - | - | - | - | - | - | - | - | - | - | - |
| **All head impacts** | | **16**  **(44.4)** | **10**  **(27.8)** | **5**  **(13.9)** | **4**  **(11.1)** | **-** | **1**  **(2.8)** | **31**  **(86.1)** | **5**  **(13.9)** | **4**  **(11.1)** | **18**  **(50.0)** | **14**  **(38.9)** | **15** |
| - Unintentional ball-to-head impacts | | - | - | 3  (75.0) | 1  (25.0) | - | - | 2  (50.0) | 2  (50.0) | - | 1  (25.0) | 3  (75.0) | - |
| - Head-to-head impacts | | - | 1  (100.0) | - | - | - | - | 1  (100.0) | - | - | 1  (100.0) | - | - |
| - Upper limb-to-head impacts | | 14  (60.9) | 6  (26.1) | - | 2  (8.7) | - | 1  (4.3) | 23  (100.0) | - | 4  (17.4) | 13  (56.5) | 6  (26.1) | 11  (47.8) |
| - Lower limb-to-head impacts | | 2  (50.0) | 1  (25.0) | 1  (25.0) | - | - | - | 2  (50.0) | 2  (50.0) | - | - | 4  (100.0) | 1  (25.0) |
| - Ground-to-head impacts | | - | 2  (66.7) | - | 1  (33.3) | - | - | 3  (100.0) | - | - | 3  (100.0) | - | 3  (100.0) |
| - Other head impacts | | - | - | 1  (100.0) | - | - | - | - | 1  (100.0) | - | - | 1  (100.0) | - |
| *Yes sanction includes fouls, first yellow card, second yellow card and straight red card given by referee | | | | | | |  |  |  |  |  |  |  |

Supplementary Table 3. Activity characteristics of potential head injuries

| **Type of head contact event** |  | | **Ball delivery method** | | | | | | | | |
| --- | --- | --- | --- | --- | --- | --- | --- | --- | --- | --- | --- |
|  | **Corner** | **Free kick** | | **Free play** | **Goalkeeper block** | **Goalkeeper kick** | **Goalkeeper throw** | **Kick in** | **Shot on goal (on target)** | **Shot on goal (off target)** | **No ball involved** |
| **All head contact events** | **-** | **-** | | **27**  **(71.1)** | **-** | **1**  **(2.6)** | **1**  **(2.6)** | **-** | **-** | **-** | **9**  **(23.7)** |
| - Total header | - | - | | 1  (50.0) | - | - | 1  (50.0) | - | - | - | - |
| - *Controlled headers* | - | - | | 1  (100.0) | - | - | - | - | - | - | - |
| - *Uncontrolled headers* | - | - | | - | - | - | 1  (100.0) | - | - | - | - |
| - *Headers with unsure control* | - | - | | - | - | - | - | - | - | - | - |
| **All head impacts** | **-** | **-** | | **26**  **(72.2)** | **-** | **1**  **(2.8)** | **-** | **-** | **-** | **-** | **9**  **(25.0)** |
| - Unintentional ball-to-head impacts | - | - | | 4  (100.0) | - | - | - | - | - | - | - |
| - Head-to-head impacts | - | - | | 1  (100.0) | - | - | - | - | - | - | - |
| - Upper limb-to-head impacts | - | - | | 14  (60.9) | - | 1  (4.3) | - | - | - | - | 8  (34.8) |
| - Lower limb-to-head impacts | - | - | | 4  (100.0) | - | - | - | - | - | - | - |
| - Ground-to-head impacts | - | - | | 2  (66,7) | - | - | - | - | - | - | 1  (33.3) |
| - Other head impacts | - | - | | 1  (100.0) | - | - | - | - | - | - | - |
| *IR: Incidence rate per 1000 match hours  **Data are mean values ± standard deviations.  *** Mean per match per player is calculated based on players on the pitch | | | | | | | | | | | |
